# Evaluation of Two New Membrane-Based and Microtiter Plate Enzyme-Linked Immunosorbent Assays for Detection of Campylobacter jejuni in Stools of Bangladeshi Children

**DOI:** 10.1128/JCM.00702-18

**Published:** 2018-08-27

**Authors:** Amanda E. Schnee, Rashidul Haque, Mami Taniuchi, M. Jashim Uddin, William A. Petri

**Affiliations:** a Division of Infectious Diseases and International Health, University of Virginia, Charlottesville, Virginia, USA; b International Centre for Diarrhoeal Disease Research, Bangladesh, Dhaka, Bangladesh; Brigham and Women's Hospital

**Keywords:** Campylobacter, ELISA, Quik Chek, diagnostic

## Abstract

Two new monoclonal antibody-based, sandwich enzyme immunoassays (EIAs) for fecal antigen detection of Campylobacter jejuni or Campylobacter coli were evaluated using diarrheal stool specimens from a cohort of children in Bangladesh. These children routinely harbor multiple enteric pathogens, often at levels that make it difficult to assign diarrheal symptoms to a causative agent. A panel of 158 PCR-positive specimens with a broad range of C. jejuni/C. coli DNA cycle threshold (*C_T_*) values was used to assess the ability of the two tests to detect C. jejuni/C. coli antigen amounts that varied widely. A panel of 100 C. jejuni/C. coli PCR-negative specimens was used to verify that the assays correctly identified specimens as negative when the sample contained other enteric pathogens. Further analysis was conducted on a subset of 46 specimens that contained particularly substantial amounts of C. jejuni/C. coli (*C_T_* of ≤19.7) that previous studies have ascribed as “diarrhea-associated.” The Quik Chek rapid EIA and the Chek enzyme-linked immunosorbent assays (ELISAs) had a sensitivity of 95.7% for these specimens (specificities, 97% and 96%, respectively), supporting the usefulness of the new Chek and Quik Chek assays in symptomatic presentations, where Campylobacter is the likely etiology.

## Abstract (precis)

Two new monoclonal antibody-based, sandwich enzyme immunoassays (EIAs) for fecal antigen detection of Campylobacter jejuni or Campylobacter coli were evaluated using diarrheal stool specimens from a cohort of children in Bangladesh. These children routinely harbor multiple enteric pathogens, often at levels that make it difficult to assign diarrheal symptoms to a causative agent.

## INTRODUCTION

Diarrhea is a major cause of morbidity and mortality worldwide (1, 2). Campylobacter jejuni and Campylobacter coli are major contributors to diarrheal illness and are currently recognized as the most common causes of foodborne gastroenteritis (3). C. jejuni and C. coli are Gram-negative bacteria which are frequently commensal organisms in birds or other mammals; thus, infection in humans is often associated with consumption of contaminated food or water (4). The resulting illness is characterized by fever, abdominal pain, and dysentery and can be difficult to distinguish clinically from infections such as shigellosis (4). Although campylobacteriosis is typically self-limited, it can result in devastating illness for the immunocompromised and can be associated with postinfectious sequelae such as reactive arthritis and Guillain-Barre syndrome (4). Furthermore, if gastroenteritis by a Shiga toxin-producing Escherichia coli infection is incorrectly diagnosed as Campylobacter, antibiotic treatment may put the patient at risk for hemolytic uremic syndrome (5, 6). Thus, there are important implications for identifying campylobacteriosis so that appropriate therapy may be initiated.

Microscopy and stool culture have historically been the gold standard for diagnosis of Campylobacter infection (4). However, PCR testing is gaining popularity as Campylobacter can prove difficult to culture and may take several days to provide a reportable result. Yet PCR may not be a viable diagnostic in resource-limited areas. Given the rate of Campylobacter infections, the possible severity of illness, and the risk of subsequent complications, there is a need for rapid and reliable testing, especially in areas with limited resources.

The Campylobacter Quik Chek rapid test and Campylobacter Chek Enzyme-Linked Immunosorbent Assay (ELISA) (TechLab, Inc., Blacksburg, VA) are newly developed testing platforms which qualitatively detect a Campylobacter jejuni/C. coli-specific antigen in human fecal specimens. The Quik Chek test is a cassette that contains a membrane with one control stripe and one stripe of test antibody. The control stripe is a rabbit anti-goat line that binds only the polyclonal goat antibody horseradish peroxidase (HRP) conjugate. The test stripe contains monoclonal antibody against a Campylobacter-specific antigen and captures antigen and antigen-conjugate complexes that are formed when HRP-conjugated anti-Campylobacter polyclonal antibody is added to the fecal sample. Addition of HRP substrate produces a visible line, which is read by eye without the need for a spectrophotometer. The Chek ELISA is a microtiter plate-based assay which qualitatively detects a Campylobacter jejuni/*C. coli*-specific antigen in stool. The wells use an antibody sandwich design similar to that of the Quik Chek. The test begins with a diluted stool sample, which binds monoclonal antibodies coated in the well that are then sandwiched by an HRP-conjugated polyclonal antibody for a visual positive result that is analyzed by machine. These tests were designed to be user-friendly and reliable and to allow rapid analyses for clinical specimens.

However, the ease of use and accuracy of these kits should also commend their application to investigations in settings of high population density and poor water sanitation, where Campylobacter is likely to be present but where multiple pathogens can often be found in a single stool sample. This study was conducted with specimens collected from a cohort of children, ages 0 to 24 months, living in the Mirpur area of Dhaka, Bangladesh. By the time they are 12 months old, children in the Mirpur community who have diarrhea are infected with five to six enteric pathogens (attack rate of 4.7 episodes per child per year); even infants without diarrhea are often infected with three enteric pathogens (7, 8). Given the similarity in clinical presentations between many diarrheal pathogens, it is often difficult to determine which organism may be contributory. Identifying a causal agent is important because treatments for enteric pathogens can vary widely and at times can be contradictory. A recent study in Vietnam found that empirical antibiotic treatment of children with acute diarrhea produced no clinical benefit and was associated with high rates of antibiotic resistance (9). Correct diagnosis is therefore necessary for correct treatment.

The present study was conducted in three phases to challenge the specificity of the new assays in the face of multiple pathogens but where campylobacteriosis was expected to be the primary diagnosis. The first phase utilized PCR-positive specimens selected to present a broad range of cycle threshold (*C_T_*) values to establish the range of C. jejuni/C. coli amounts that the two tests could detect. The second phase used a panel of PCR-negative specimens to establish how well the tests could correctly identify specimens as negative when the stool contained other enteric pathogens. The third phase analyzed Campylobacter PCR-positive specimens with *C_T_* values indicating that C. jejuni/C. coli was sufficiently abundant in the stool to be the probable cause of the diarrheal symptoms.

## MATERIALS AND METHODS

The data presented here represent the first time the Campylobacter Quik Chek and Campylobacter Chek ELISA kits have been tested with a large cohort of pediatric patient specimens from a low-resource setting. All diarrheal stool samples were collected from a cohort of children, ages 0 to 24 months, living in the Mirpur area of Dhaka, Bangladesh, where pediatric Campylobacter infection is highly prevalent (8, 10). The specimens were collected in Mirpur and housed at the International Centre for Diarrheal Disease Research, Bangladesh (icddr,b), prior to shipment to Charlottesville, VA, for testing. The samples were stored, on average, for 5 years at −80°C until testing was performed. Quantitative PCR (qPCR) testing via custom TaqMan Array Cards (TAC) was performed on all diarrheal stool samples at the time of collection, as part of the analysis done during the original PROVIDE (Performance of Rotavirus and Oral Polio Vaccines in Developing Countries) study (11).

Previously recorded analytical *C_T_* values were used to assign which samples were positive for C. jejuni/C. coli DNA. Thus, specimens with *C_T_* values of <35 were classified as being positive for C. jejuni/C. coli DNA, those with *C_T_* values of ≥35 were classified as being negative. The TAC tests also determined if DNA from 19 other pathogens was present in these samples, using the same *C_T_* cutoffs for positivity. Although *C_T_* values were available for Campylobacter 16S rRNAs from a broad array of species, these data were not analyzed as previous studies had shown that 16S PCR also detected unusual Campylobacter species that were present in specimens from the Mirpur area and that were not expected to be detected by the enzyme immunoassays (EIAs) (12). From just under 2,000 available diarrheal stool samples, 542 were positive for Campylobacter; of these 158 specimens with C. jejuni/C. coli *C_T_* values of <35 and 100 specimens with C. jejuni/*coli C_T_* values of ≥35 were randomly selected for antigen testing. Results were obtained from the TechLab Campylobacter Quik Chek test and Campylobacter Chek ELISA.

Another study whose sites included Bangladesh, the Global Enteric Multicenter Study (GEMS), utilized custom qPCR cards with Campylobacter primers of the same design and sensitivity (13). That study defined a cycle threshold (*C_T_*) value of ≤19.7 as an amount of Campylobacter that was likely to be responsible for the patient's current illness, despite the presence of other potential diarrheal pathogens. This *C_T_* value was designated the “diarrhea-associated quantity.” We separately analyzed the results of the Quik Chek and Chek ELISAs on the subset of samples with these large amounts of Campylobacter.

### Campylobacter Quik Chek.

Stool samples were brought to room temperature before testing was initiated. Required reagents for the Quik Chek test were supplied either in bottles with droppers with graduated markings or in tipped bottles to allow for rapid and easy measurement. Stool sample (25 μl) was transferred via pipette into a specimen dilution tube containing 750 μl of diluent and 50 μl of conjugate. The specimen dilution tube was then vortexed for 15 s to ensure adequate suspension. Diluted sample (500 μl) was loaded into the sample window of the cassette. Cassettes were incubated at room temperature for 15 min, following which 300 μl of wash buffer was loaded into the reading window. Wash buffer was allowed to absorb entirely; then 2 drops of substrate was loaded into the reading window. The cassettes were again incubated at room temperature for 10 min, and results were read visually immediately following the incubation period. A specimen was interpreted as positive if both test and control lines were present (see Fig. S1 in the supplemental material). The color of the lines ranged from dark blue to light blue. A sample was interpreted as negative if only the control line was visible. The test was invalid if the control line was absent.

### Campylobacter Chek ELISA.

Stool samples were brought to room temperature before testing was initiated. Required reagents for the ELISA were supplied in either bottles with measured droppers or tipped bottles to allow for counted drops to be applied to the wells. For the Campylobacter Chek test, 50 μl of stool sample was transferred via pipette into a specimen dilution tube containing 200 μl of diluent. The specimen dilution tube was then vortexed for 15 s to ensure adequate suspension. Diluted sample (100 μl) and 1 drop (50 μl) of conjugate were loaded into each well. The plate was then sealed and incubated for 50 min at 37°C without shaking. Wells were rinsed with wash buffer five times, and the plate was struck hard against a paper towel-covered surface between washes to fully remove any remaining fecal particulates. Any remaining visible particulate mandated an additional wash cycle. Following washing, 2 drops of substrate was added to each well, and the plate was incubated at room temperature for 10 min. A drop of stop solution was then added to each well; the plate was incubated at room temperature for a final 2 min and then was ready for immediate analysis. Plates were read using a BioTek Synergy H4 hybrid microplate reader (BioTek Instruments, Inc., Winooski, VT) at an optical density (OD) of 450 nm blanked against an OD of 620 nm, and values greater than 0.08 were considered positive. For these samples, OD values ranged from 0.08 to 3.7.

## RESULTS

We tested 158 pediatric diarrheal stool samples that had initially been identified by PCR as positive for C. jejuni/C. coli DNA. Tables 1 and 2 show the results of testing from both the Chek and the Quik Chek assays. The *C_T_* value assigned as positive in the original PROVIDE data set was a *C_T_* of <35. Notably, the samples from the entire PROVIDE cohort of 543 Campylobacter-positive specimens presented a continuum of *C_T_* values, with no evidence of a peak where positive specimens were more abundant (see Fig. S2A and B in the supplemental material). This indicated that specimens from patients with diarrhea contained a very wide range of Campylobacter DNA amounts that were clinically relevant and that there was no helpful discriminatory gap that clearly defined positive and negative specimens. The *C_T_* values for the 158 selected samples ranged from 15.49 to 34.97 (Fig. S2B). Fifty-one samples with *C_T_* values of <20 and 55 samples with *C_T_* values between 30 and 35 were selected to provide full coverage of these clinically important ranges. A further 52 samples were selected with *C_T_* values between 20 and 30.

**TABLE 1:** Rates of positive test results for the TechLab Campylobacter Quik Chek and TechLab Campylobacter Chek ELISA for diarrheal stool samples with various amounts of Campylobacter jejuni/C. coli

C. jejuni/C. coli sample type (*C_T_* value)	No. of samples	No. of positive results (%)	
		Quik Chek	Campylobacter Chek
qPCR positive (<35)	158	121 (76.6)	123 (77.8)
qPCR negative (≥35)	100	3 (3)	4 (4)
Positive at DAQ (≤19.7)^*a*^	46	44 (95.7)	44 (95.7)

a DAQ, diarrhea-associated quantity.

**TABLE 2:** Comparison of the performance characteristics of the TechLab Campylobacter Quik Chek and TechLab Campylobacter Chek ELISA^*a*^

Positive-sample *C_T_* value and test^*b*^	Sensitivity (%)	Specificity (%)	PPV (%)	NPV (%)
19.7 > *C_T_* < 35 (*n* = 112)				
Quik Chek	87.5	97	96.3	72.4
Campylobacter Chek	70.5	96	95.2	73.3
*C_T_* ≤ 19.7 (*n* = 46)^*c*^				
Quik Chek	95.7	97	93.6	98
Campylobacter Chek	95.7	96	91.7	98

a Test results are for samples in which C. jejuni was present above and below diarrhea-associated *C_T_* values. PPV, positive predictive value; NPV, negative predictive value.

b For both groups, data include results for 100 negative samples with *C_T_* values of ≤35.

c The *C_T_* value represents the diarrhea-associated quantity.

The Chek ELISA and the Quik Chek assays could identify specimens as positive throughout this wide range of DNA concentrations. As might be expected, the percentage of samples remaining EIA antigen-positive increased as the *C_T_* value decreased. Below a *C_T_* value of 30, the Quik Chek and Chek ELISAs identified 88% and 91% of the specimens, respectively, as positive, while above a *C_T_* value of 30 the positivity rates were 55% and 53%. To further assess clinical relevance of these new assays, samples for which *C_T_* values were less than <19.7 were considered separately. This *C_T_* value, the diarrhea-associated quantity, was obtained from the GEMS, which analyzed stool samples from children with moderate to severe diarrhea and from their age-/sex-matched controls to identify the etiologies of diarrhea at seven different sites across Africa and Asia (13). Quantitative PCR was performed on all stool samples collected, and the quantity of a pathogen felt to be causal of an episode of diarrhea was defined based on the point at which the 95% confidence interval of the odds ratio between cases and controls was greater than 1 (13). The GEMS determined this parameter for 19 different enteric bacteria and viruses. For C. jejuni/C. coli, the diarrhea-associated *C_T_* value was 19.7. This *C_T_* value equates to approximately 2.5 × 10^7^ copies of C. jejuni/C. coli DNA per gram of stool (13). Among our 158 samples, there were 46 for which the *C_T_* value of C. jejuni was 19.7 or lower. The Quik Chek and the Chek ELISAs detected 44 of these (Table 1). In addition, 100 specimens considered negative for C. jejuni/C. coli, with *C_T_* values not enumerated but classified as ≥35, were tested. The Quik Chek identified three specimens as positive (3%) while the Chek ELISA identified four specimens as positive (4%).

## DISCUSSION

Diagnosis of diarrheal diseases is challenging in many resource-limited environments. The pathogen burden of children in environments with poor sanitation can be extraordinary, and colonization/infection appears to begin within weeks of birth. Even nondiarrheal specimens may contain enteropathogens. A median of three pathogens has been found in specimens from symptom-free Bangladeshi children. In diarrheal specimens, coinfections of two or more pathogens were found in 96.2% of the children (7).

For Campylobacter, the traditional diagnostic test is culture. However, C. jejuni/C. coli bacteria are microaerophilic and can die erratically when exposed to air during specimen transport from field collection sites to the laboratory and during storage prior to testing (14). Even if a sample can be obtained from patients who present for care, culture requires 2 to 3 days for results, thus necessitating either immediate empirical treatment or an unlikely return visit (4, 12, 15). In addition, several studies indicate that other, difficult-to-culture species of Campylobacter (Campylobacter hyointestinalis, Campylobacter troglodytis, and Campylobacter upsaliensis) can be present in specimens collected in multiple resource-limited settings and that C. jejuni and C. coli may represent less than half of all infections (12, 15, 16). Given these limitations of culture, it is not surprising that culture-independent diagnostic tests for Campylobacter have found prevalence rates that are higher than have been formerly recognized (17). This suggests that actual disease caused by Campylobacter, certainly in low-resource settings and perhaps in developed countries as well, is more common than previously appreciated.

The Campylobacter Quik Chek and Campylobacter Chek ELISAs were designed for rapid analysis of clinical samples coupled with ease of administration. By detecting a Campylobacter-specific antigen, these tests no longer rely on bacterial viability, nor do they require specialized growth media and technicians trained to identify the pinpoint colonies of Campylobacter among competing fecal flora. These tests are user-friendly and require only a short amount of time to run to completion: less than 30 min for the Quik Chek assay and less than 60 min for the Chek ELISA. A rapid time-to-result for a positive specimen would provide a clinician more confidence to recommend antibiotic treatment of the patient and to begin such treatment without delay. Further, both assays are easily portable, as all required materials fit comfortably into small packaging and require only ice packing for transport. The Quik Chek assay would be especially useful in resource-limited settings as it requires no further instrumentation for interpretation. In addition, fresh samples could be stored at 2 to 8°C for up to 96 h after collection or be frozen below−10°C and thawed up to 5 times (sample handling recommendations per kit manufacturer). The samples tested in this study had been stored at −80°C for up to 5 years and still showed good antigen reactivity.

The 158 positive specimens tested in this study were derived from patients with diarrheal symptoms and thus encompass the range of clinically relevant pathogen burden. However, it became clear that the amounts of C. jejuni/C. coli in these samples varied extensively. The Campylobacter Quik Chek and Campylobacter Chek ELISAs could detect the presence of C. jejuni/C. coli over this entire span.

Most notably, however, both assays performed with improved efficacy when testing samples in which C. jejuni/C. coli was present in quantities that made Campylobacter the probable cause of the patient's diarrhea, i.e., samples where the *C_T_* value for C. jejuni/C. coli was at or below the diarrhea-associated quantity (Table 2). Both the Campylobacter Quik Chek and Campylobacter Chek ELISAs had improved sensitivity (>95%) and negative predictive value (98%) when used in these settings, reinforcing the ability of these tests to correctly determine not only when Campylobacter is present but also when it is more likely to be a causal organism as opposed to asymptomatic carriage.

**FIG 1:**
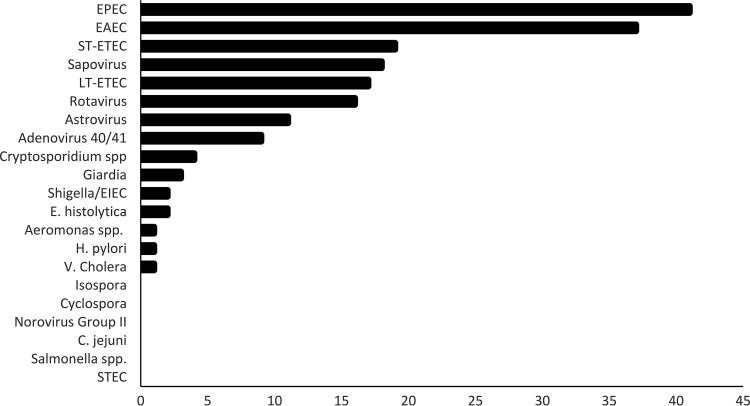
Frequency of detections in stool samples, by pathogen, for C. jejuni-negative samples. Abbreviations: EPEC, enteropathogenic E. coli; EAEC, enteroaggregative E. coli; ST-ETEC, heat-stabile toxin-producing enterotoxigenic E. coli; LT-ETEC, heat-labile toxin-producing enterotoxigenic E. coli; EIEC, enteroinvasive E. coli; STEC, Shiga toxin-producing enterotoxigenic E. coli.

**FIG 2:**
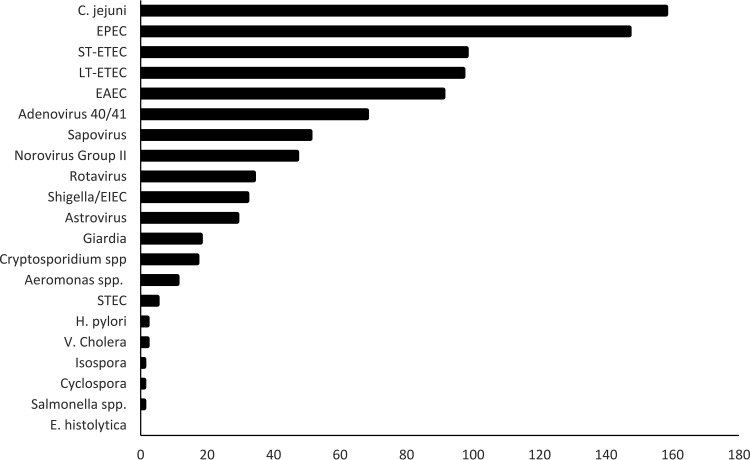
Frequency of detections in stool samples, by pathogen, for C. jejuni-positive samples. Abbreviations: EPEC, enteropathogenic E. coli; ST-ETEC, heat-stabile toxin-producing enterotoxigenic E. coli; LT-ETEC, heat-labile toxin-producing enterotoxigenic E. coli; EAEC, enteroaggregative E. coli; EIEC, enteroinvasive E. coli; STEC, Shiga toxin-producing enterotoxigenic E. coli.

Last, among 100 specimens that were designated Campylobacter negative, the Campylobacter Quik Chek and Campylobacter Chek ELISAs showed a less than 4% false-positive rate and discriminated the inferred absence of C. jejuni/C. coli in specimens that contained an average of two other enteropathogens, including bacteria, viruses, and protozoa (Fig. 1 and 2). Analytical studies of various amounts of C. jejuni spiked into Campylobacter-negative stool indicated that the Campylobacter Quik Chek assay has a limit of detection of 8.4 × 10^4^ CFU/ml feces (95% confidence interval [CI], 7.37 × 10^4^ to 9.97 × 10^4^ CFU/ml), and the Campylobacter Chek ELISA has a detection limit of 2.1 × 10^5^ CFU/ml (95% CI, 1.89 × 10^5^ to 2.4 × 10^5^ CFU/ml). These are the lowest limits available for an FDA-cleared Campylobacter immunoassay. Unfortunately, for specimens with *C_T_* values near 35, it was not possible, in the face of up to six pathogens with potential to cause diarrheal symptoms, to know whether the detected C. jejuni/C. coli contributed to the patient's distress.

Taken together, the Campylobacter Quik Chek and Campylobacter Chek ELISAs are effective testing methodologies for situations in which ease of test administration/interpretation and rapidity of diagnosis would be paramount. Both assays would provide improved Campylobacter diagnosis in settings where causality is in question so that therapy may be appropriately directed.

## Supplementary Material

Supplemental file 1
