# Supplementary material for: Evaluation of Two New Membrane-Based and Microtiter Plate Enzyme-Linked Immunosorbent Assays for Detection of Campylobacter jejuni in Stools of Bangladeshi Children
Source: J Clin Microbiol. 2018 Aug 27;56(9):e00702-18. doi: 10.1128/JCM.00702-18 (PMC6113481; doi:10.1128/JCM.00702-18)

Supplementary Material

Figure 1S: *Campylobacter* QUIK CHEK™ for the detection of *C. jejuni* in stool samples. The cassette on the left is an example of a negative result, showing only the control line in the reading window. The cassette on the right is an example of a positive result, showing both the control line to the left side of the reading window and the positive result line on the right side of the reading window.

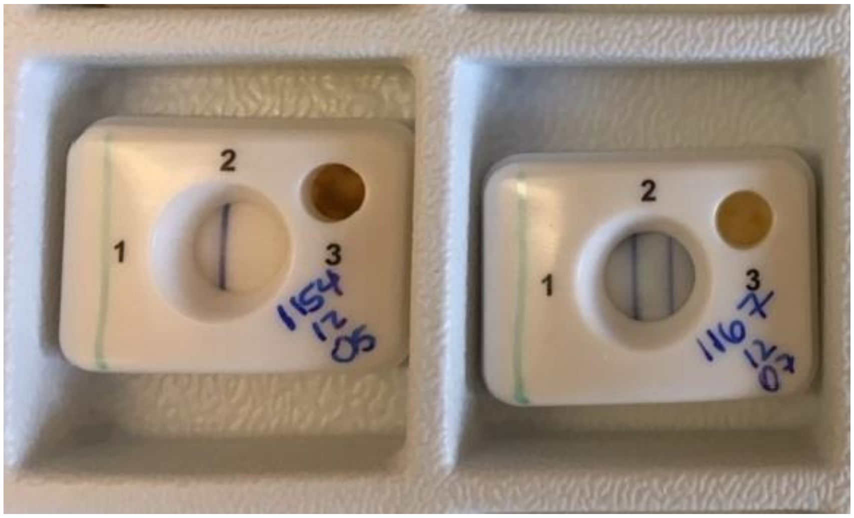

Figures 2SA and 2SB: Ct values for the 158 *C. jejuni* positive stool samples tested in the current study (A) and all 542 *C. jejuni* positive stool samples in the PROVIDE cohort (B).

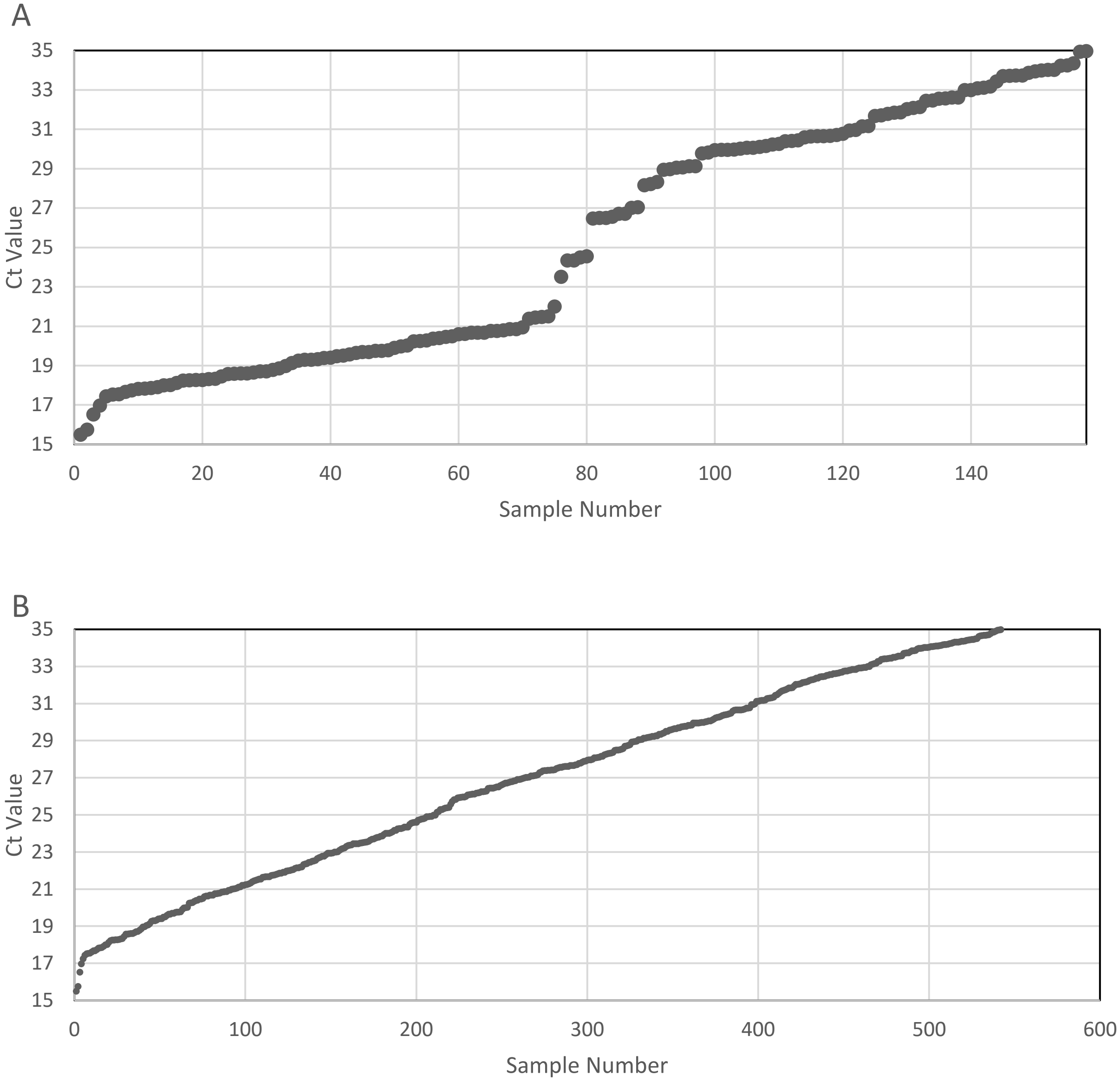

Supplement: Supplemental file 1 [file zjm999096075s1.pdf]
